# Web discussions on cardiovascular diseases: pre-COVID-19 evaluation and impact of the COVID-19 pandemic – Web listening analysis in the Italian population

**DOI:** 10.1186/s12889-024-20615-5

**Published:** 2024-11-28

**Authors:** Isabella Cecchini, Matteo Biroli, Katia Massaroni, Emanuela Folco, Maria Rita Montebelli, Giuseppe Ciancamerla, Daniela Giudice, Roberto Franco Enrico Pedretti, Paolo Magni

**Affiliations:** 1 Primary Market Research, IQVIA, Via Fabio Filzi 29, Milan, 20124 Italy; 2 Università degli Studi di Milano, Via Festa del Perdono 7, Milan, 20122 Italy; 3 General Medicines Medical Department, Sanofi, Viale Luigi Bodio 37/b, Milan, 20158 Italy; 4 Fondazione Italiana per il Cuore, Viale Piave 35, Milan, 20129 Italy; 5 Dipartimento Scienze Mediche e Chirurgiche, Fondazione Policlinico Universitario Agostino Gemelli IRCCS, Largo Agostino Gemelli 8, Rome, 00168 Italy; 6 Conacuore, Via Zurlini, 130, Modena, 41125 Italy; 7 Dipartimento Cardiovascolare, IRCCS MultiMedica, via Milanese 300, Sesto S. Giovanni, Milan, 20099 Italy; 8 School of Medicine and Surgery, University of Milano Bicocca, Piazza dell’Ateneo Nuovo 1, Milan, 20126 Italy; 9 Dipartimento di Scienze Farmacologiche e Biomolecolari, Università degli Studi di Milano, via Balzaretti 9, Milan, 20133 Italy; 10 IRCCS MultiMedica, via Milanese 300, Sesto S. Giovanni, Milan, 20099 Italy

**Keywords:** Social media listening, Web discussion, Cardiovascular disease, COVID-19, Patient insight, Prevention

## Abstract

**Background:**

Web discussions on health issues are becoming very relevant in the general public. In this context, little information is available regarding cardiovascular diseases, which remain the first cause of morbidity, disability and mortality worldwide. The central objective of the study was to conduct a Web listening analysis on discussions about cardiovascular diseases in Italy, comparing the data relative to the 2-year pre-COVID-19 pandemic period with those collected during the COVID-19 pandemic lockdown (March-July 2020), with quantification of conversations on cardiovascular disease and Web-based discussions and specific evaluation of COVID-19 lockdown impact.

**Methods:**

A retrospective Web listening analysis using publicly available data was conducted, using validated methods that allow to estimate cardiovascular disease awareness. Digital sources were identified to retrieve data (Italian language), relevant to cardiovascular disease topics. Data were analysed by Google Trends methodology and the Digital Intelligence Platform Brandwatch. Natural Language Processing algorithms enabled comparative analysis, topic detection, classification, leading to a 279,790-item dataset.

**Results:**

News channels and Twitter were the most important platforms feeding cardiovascular disease information. Facebook was mostly relevant for information sharing. In the pre-COVID-19 period, cardiovascular disease ranked 5th among main health issues (vaccines, tumors, influenza, diabetes) on the Web, and the most discussed cardiovascular disease themes were symptoms/diagnosis (34%), treatments (20%), disease causes/triggers (11%), disease information (9%), quality of life (8%). Conversations on cardiovascular disease prevention were marginal (5%). The COVID-19 pandemic lockdown strongly impacted on discussed topics; novel themes emerged: hospitalization, death risk/occurrence, greater cardiovascular disease risk. Discussions on cardiovascular disease prevention remained marginal (4%). COVID-19 pandemic increased fear of severe COVID-19 among patients with cardiovascular disease and worsened quality of relationship/contact with physicians.

**Conclusions:**

A limited awareness of cardiovascular disease and their prevention was observed before and during the COVID-19 pandemic. Patients/caregivers need more information and contact with physicians, as it emerged during COVID-19 pandemic. It is urgent to promote novel prevention strategies and to engage people leveraging digital channels and social media.

**Graphical Abstract:**

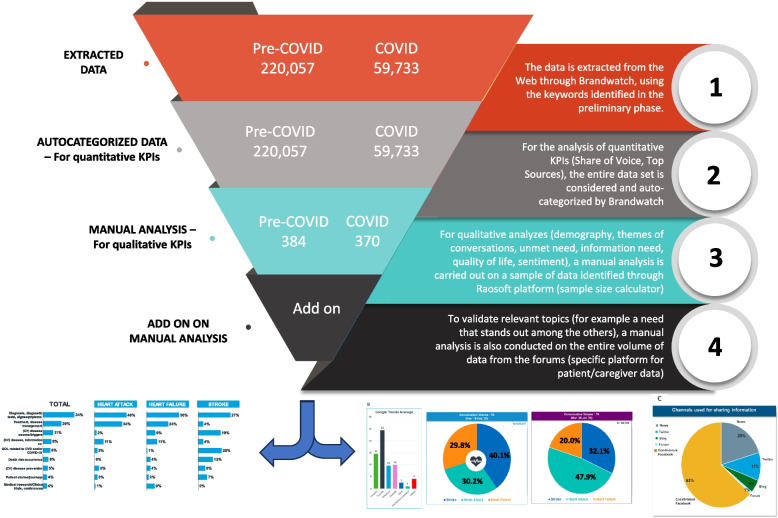

**Supplementary Information:**

The online version contains supplementary material available at 10.1186/s12889-024-20615-5.

## Background

Cardiovascular diseases (CVD) account for half deaths related to noncommunicable disease (NCD) globally [1]. Prior to the COVID-19 pandemic, [2] CVD were the leading cause of death in almost every world region [1]. Many risk factors promoting CVD are preventable, including behavioral factors (tobacco and alcohol abuse, unhealthy diets, physical inactivity, etc.), and modifiable clinical factors (arterial hypertension, obesity, dyslipidemia, type 2 diabetes mellitus and others) [2]. Therefore, increasing the awareness about these issues among citizens is pivotal for any effective primary and secondary prevention of cardiometabolic diseases [3, 4]. However, current awareness and empowerment level about different CVDs is rather low worldwide, specifically concerning stroke, heart attack [5–9] and hypertension [10, 11]. Since the beginning of the COVID-19 pandemic, the global attention on disease burden has largely been focused on communicable diseases rather than CVD and other NCDs [12–14], with relevant redirection of most resources to deal with the COVID-19 emergency [15–18]. CVDs in any case play an important role in the development of severe COVID-19 and related mortality [19, 20]. Moreover, some impact on CVD risk could also be associated to a larger diffusion of unhealthy habits due to lockdown implementation [21, 22]. It is then important to assess how the COVID-19 pandemic affected social communication and perception towards the different health issues by the general population, possibly in comparison with the pre-COVID-19 situation [11]. To implement locally effective strategies, it is relevant to accurately profile their actual extent on a regional basis, and leverage the current communication channels, with reference to the Web as source of information. The data obtained are useful for planning specific actions by stakeholders involved in the management of individual and social health. Web (social media) listening is the process of identifying and assessing what is being said and discussed about a certain topic on the Internet. It is a social approach useful for understanding Internet-based discussions among the general public and health care professionals (HCPs) on CVD awareness and public knowledge on prevention and treatment. The recently-developed analysis of the discussions on the Internet and social networks is an innovative health-information-gathering technique [23], providing unique insights into what citizens/patients actually know and think about diseases and treatments. The information and opinions shared on the Internet can be considered direct, genuine, and unprompted, offering access to patients’ conversations and can influence patient behaviors [24–27]. Within Italy, the country that has been the target of the present study, Internet use showed an important growth in the last years. In 2020, 83.7% (50 mln) of the population used the Internet [28]*.* Seven out of 10 Italians look for information on health from different sources, like general practitioners (73%), pharmacists (57%), and Internet (57%), which thus represents a substantial source of health information [29]. Given the increase in the use of the Internet and social networks to collect information on health, combined with the dramatic impact of the COVID-19 pandemic on health systems and individuals, the central objective of the current study was to implement a Web Listening analysis on CVDs discussions in Italy, comparing the pre-COVID-19 pandemic period (February 2018 – February 2020) with the first COVID-19 pandemic timeframe (March-July 2020), characterized by a dramatic infection spread and a lockdown. Specific research questions included: i) quantification of searches and conversations on CVDs; ii) analysis of the Web-based discussions on CVDs before and during the COVID-19 pandemic in Italy; iii) comparison of the topics discussed before and during the COVID-19 pandemic and assessment of the impact of the pandemic on individuals’ perceptions of CVDs.

## Methods

### Study design

We conducted a retrospective Web listening analysis using data available in the public domain. A comprehensive Web listening analysis process was developed to identify and categorize publicly available Web and social media conversations during the first timeframe (lockdown and re-opening) of the COVID-19 pandemic in Italy (March 2020 – July 2020), in comparison to the two previous years before the pandemic (February 2018 – February 2020). This type of search is not allowing easy reference to the geographical distribution of the conversations, due to the intrinsic properties of the Web. Taking this into consideration, the search was restricted to the Italian language to possibly focus the collected information relative to a clearly defined geographical area, that is Italy, a rather large country.

From the ethical standpoint, the data from the public domain were collected and analyzed taking care of the main issues of information ethics, such as privacy and anonymization, accuracy, property and accessibility. This research was conducted according to the 2021 EPHMRA Code of Conduct v. 2.11.21.

### Methodological approaches

The data were collected and analyzed by a multidisciplinary team of life science-qualified analysts (I.C., M.B.), social media experts (M.F., I.G.), and physicians (P.M.). Digital sources were identified to retrieve data in the Italian language, relevant to the selected CVD topics (stroke, heart attack, heart failure). Related to them, a robust keyword taxonomy was created to identify lexical entities in conversations related to the research objectives, including words, phrases, and hashtags. A preliminary analysis of the data indexed by prominent search engines, such as Google, was performed, through the Google Trends methodology, to compare the search frequency on the Web search engines of CVD-related keywords such as “ictus” (Italian word for stroke), “infarto” (heart attack), “scompenso cardiaco” (heart failure) with other common diseases/conditions: “tumore” (tumor/cancer), “diabete” (diabetes), “vaccini” (vaccines). Afterward, a systematic process of data collection provided by the Brandwatch software was applied, which is an automated tool that is compliant with data privacy regulations and is compliant with policies of the utilized sources, Websites and social networks. Indeed, data sources considered for user-generated content were mainly online news-channels, Twitter, Facebook, blogs, and online forums. Only publicly available information on these digital platforms were accessed and used for research and no password-restricted information was retrieved. All patient-identifiable information available on social media were de-identified at source and were not considered for analysis in the current study.

The automated data identification was completed using Digital Intelligence Platform Brandwatch. Natural Language Processing (NLP) algorithms systems enabled comparative analysis, topic detection and classification. After that, a manual check was conducted to assess the appropriateness of the data. Data from forums and blogs were then manually analyzed to identify characteristics of the discussant and assess the themes of conversations in more depth. Conversation topics by different stakeholders (including patients, caregivers, and health care professionals) were identified and analyzed by a qualitative study, using a deep dive analysis carried out by a multidisciplinary team. Information related to user demographics, such as age, sex, and geographic location of social media users, were recorded when explicitly specified by online users.

The term “Social Media Conversations” is used to identify the entire volume of data. “Mentions” refers to any type of data from any type of source – for example, news articles, tweets, comments, etc.), and the term “Discussions” refers to specific interactions between patients, caregivers or HCPs in forums and blogs, which have been used for the analysis of topics of conversation.

Based on the keywords, a set of 279,790 data linked to CVDs keywords has been collected during the entire period February 2018-July 2020 and quantitatively analyzed. The majority (*n* = 220,057; 78.7%) were in the pre-COVID-19 period and the remaining 59,733 belonged to the COVID-19 timeframe (*n* = 37,447; 62.7% in the lockdown phase, and *n* = 22,286; 37.3% in the re-opening phase) (Table 1). Afterwards, the qualitative analysis has been manually performed by the team of experts on a selected set of 758 conversations (360 conversation from pre-COVID; 398 conversations from COVID period), using the above-mentioned keyword taxonomy (Table 1).

**Table 1:** Data analysis flow

	Pre COVID-19 (February 2018—February 2020)	COVID-19 (March 2020—July 2020)	Notes
Extracted data	234,886	59,733	Data were extracted from the Web through the Brandwatch software, using the keywords selected in the preliminary phase
Autocategorized data for quantitative analysis	220,057	59,733	Data set used for the quantitative KPIs (volume of conversations, sources, reporters, geographic)
Selected data for qualitative analysis	360	398	Conversations extracted from the interactive sources (forums, blogs) used for the qualitative analysis of topics of conversations

The table shows the analysis process of data set in the 2 timeframes: Pre-COVID-19 (February 2018-February 2020) and COVID-19 (March 2020-July 2020)

*KPI* key performance indicator

## Results

### Search of health-related words on Google via Google trends

**Fig. 1:**
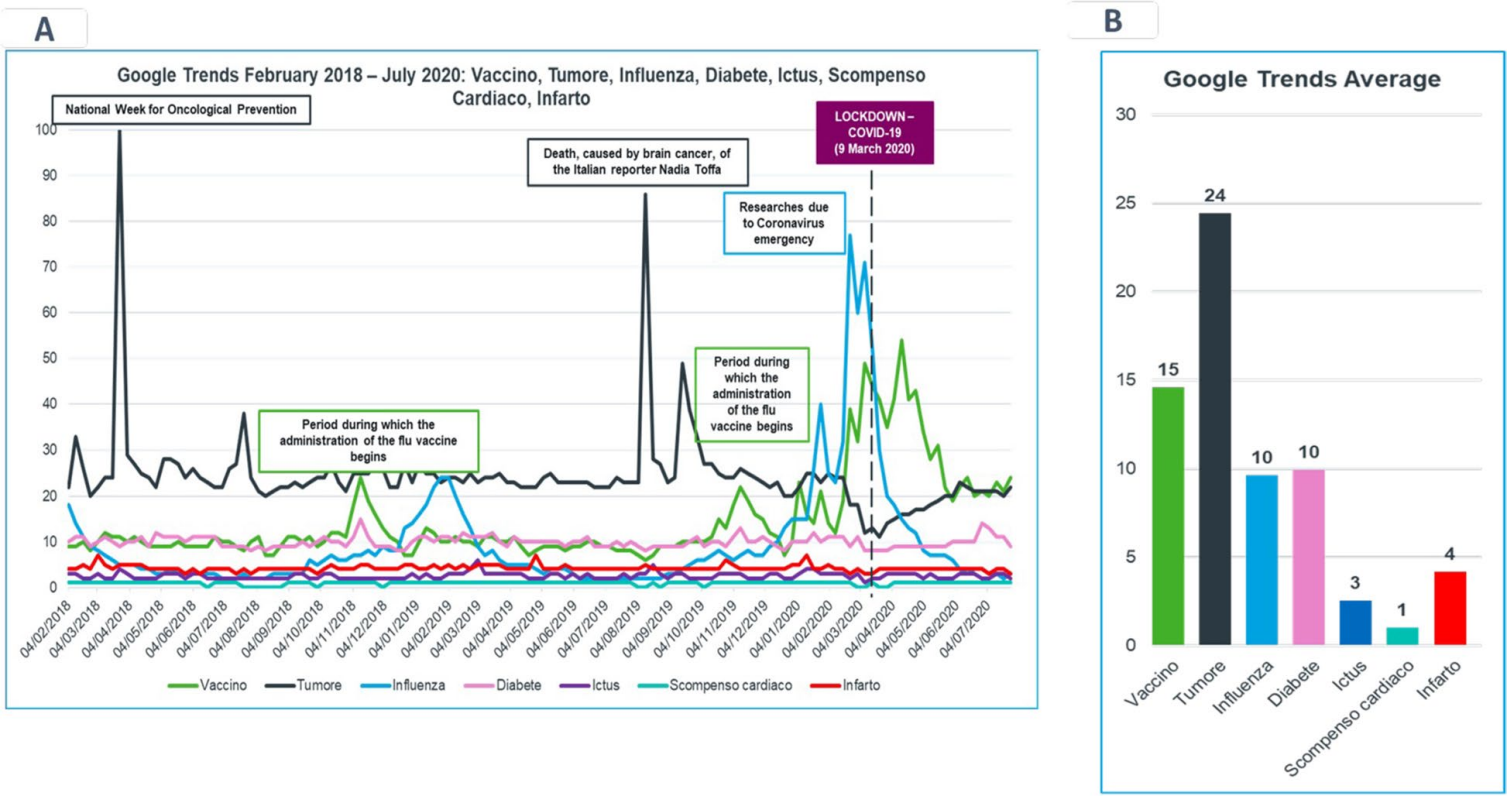
Google trends searches on 6 diseases in Italy. Panel **A** volume of searches on 6 diseases over the period February 2018- July 2020: “Vaccino” (vaccine), “Tumore” (cancer), “Influenza”, “Diabete” (diabetes), “Ictus” (stroke), “Scompenso Cardiaco” (heart failure), “Infarto” (heart attack). The Y axis shows the volume of searches for each topic considered. Google Trends normalizes search data to make comparisons between terms easier. Search results are normalized to the time and location of a query by the following process: each data point is divided by the total searches of the geography and time range, to compare relative popularity. Otherwise, places with the most search volume would always be ranked highest. The resulting numbers are then scaled on a range from 0 to 100, based on a topic’s proportion to all searches on all topics. The X axis shows the trend of searches over time. Panel **B** comparison of searches (average) during the period February 2018-July 2020 of the 6 diseases: “Vaccino” (vaccine), “Tumore” (cancer), “Influenza”, “Diabete” (diabetes), “Ictus” (stroke), “Scompenso Cardiaco” (heart failure), “Infarto” (heart attack)

According to Web analysis by Google Trends methodology, we observed that, over the whole timeframe of the analysis (February 2018- July 2020), “Tumore” (cancer) was the most searched word on Google within the set of diseases taken into consideration (vaccines, influenza, diabetes, stroke, heart failure, heart attack). However, during the COVID-19 pandemic timeframe, “Influenza” and “Vaccino” (vaccine) were the most searched words (Fig. 1A and B).

**Fig. 2:**
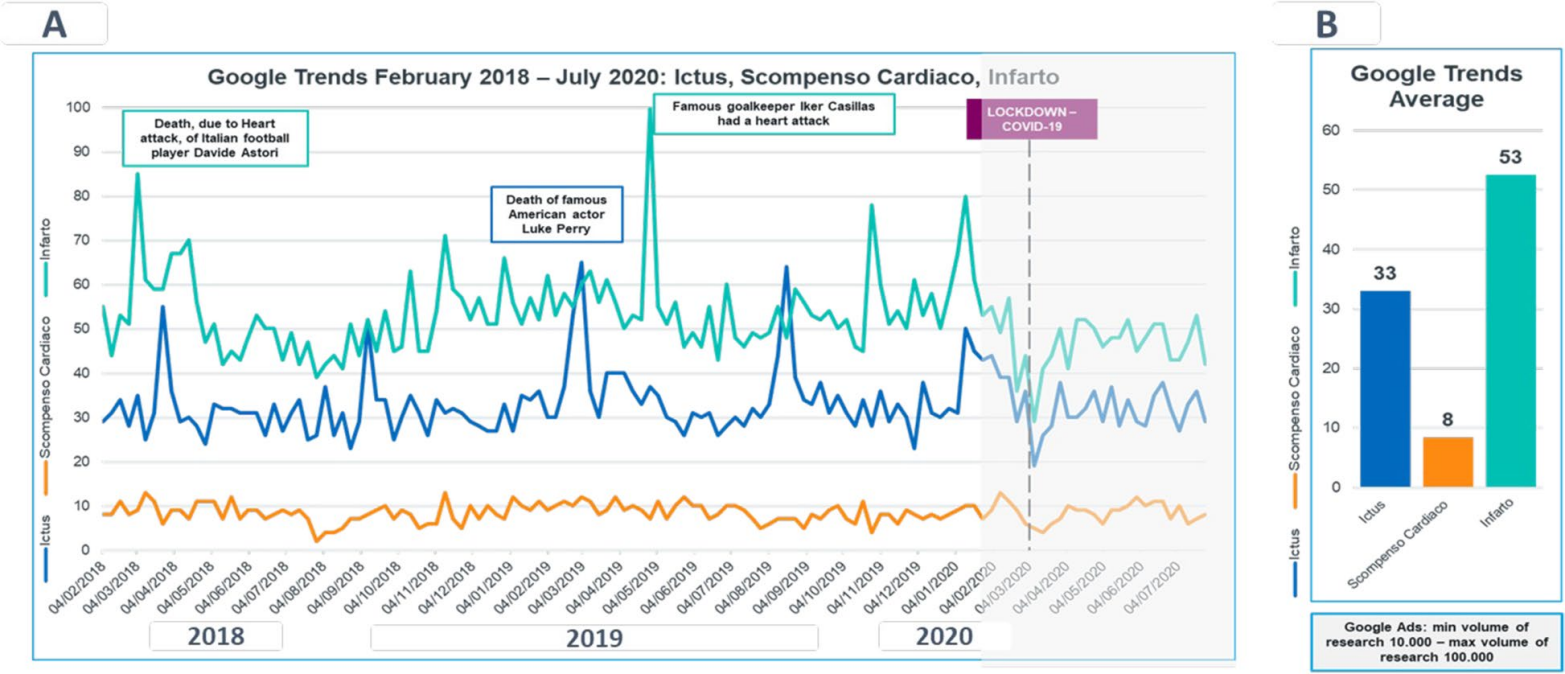
Google trends research on cardiovascular diseases in Italy. Panel **A** volume of searches in the period February 2018- July 2020 of 3 CVDs in focus: “Ictus” (stroke), “Scompenso Cardiaco” (heart failure), “Infarto” (heart attack). Peaks are linked to social events associated to the CVDs (*i.e.*death of famous actors due to hearth attack). The Y axis shows the volume of searches for each topic considered. Google Trends normalizes search data to make comparisons between terms easier. Search results are normalized to the time and location of a query by the following process: each data point is divided by the total searches of the geography and time range, to compare relative popularity. Otherwise, places with the most search volume would always be ranked highest. The resulting numbers are then scaled on a range from 0 to 100, based on a topic’s proportion to all searches on all topics. The X axis shows the trend of searches over time. Panel **B** comparison of searches (average) during the period February 2018-July 2020 of 3 CVDs in focus: “Ictus” (stroke), “Scompenso Cardiaco” (heart failure), “Infarto” (heart attack)

Within the set of CVD-related words, “Infarto” (heart attack), followed by “Ictus” (stroke), was the most searched disease term on Google. During the first week of the lockdown in Italy (beginning on March 9, 2020) and the following weeks, the trend registered a strong decline of all searches about CVD-related words on Google (Fig. 2). The graph shows the dynamics of words searched on Google, which, as expected, increased due to relevant social events or death linked to popular people (*i.e.* fatal heart attack in a famous football player).

### Analysis of volume and source of conversations related to CVD topics

**Fig. 3:**
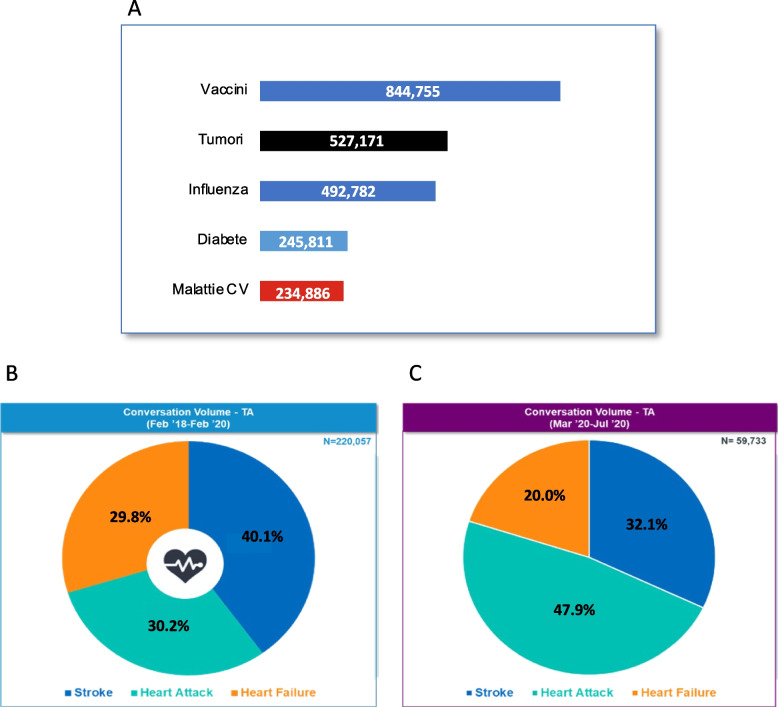
**A** Volume of conversations in the period February 2018-February 2020—Google trends searches on CV diseases:“Vaccini” (vaccines), “Tumori” (cancer), “Influenza”, “Diabete” (diabetes), “Malattie CV” (cardiovascular diseases, including stroke, heart failure, heart attack). **B** Share of conversations on stroke, heart attack and heart failure during the period February 2018- February 2020 (pre-COVID-19 period). **C** Share of conversations on stroke, heart attack and heath failure during the period March 2020 – July 2020 (COVID-19 period)

Similar to Google searches, during the period February 2018-February 2020, Web mentions associated to health topics were also primarily focused on vaccines and tumors, and discussions on CVDs ranked only at the 5th place (Fig. 3A). The volume of mentions on CVDs was comparable with that about diabetes.

**Fig. 4:**
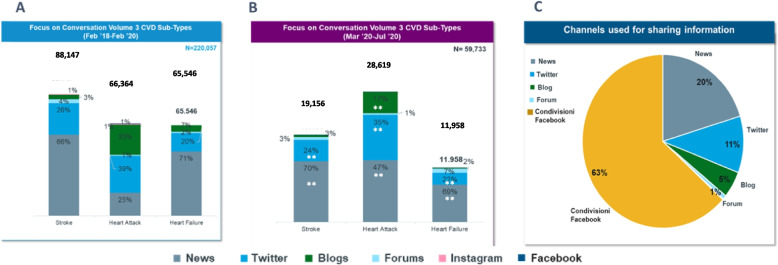
Share of sources in the pre-COVID-19 period and in the COVID-19 period. Panel **A** shows the share of Web sources of CVD conversations observed during the February-2018-February 2020 period. Panel **B** shows the share of Web sources of CVD conversations observed during the March 2020 – July 2020 period. Panel **C** Relative weight of Web sources used to share information during the February-2018-February 2020 period. *p<0.05; **p<0.01 (chi-square test; pre COVID-19 vs. COVID-19)

During the pre-COVID timeframe, mentions within the CVD area were mainly focused on stroke (40%), followed by heart attack (30%) and heart failure (30%) (Fig. 4B). During the COVID-19 pandemic timeframe, mentions on heart attacks markedly increased (47.9%), which is + 59% compared to the previous period (Fig. 3C).

### Analysis of the information sources

News channels and Twitter were found to be the most important platforms feeding information about CVDs, followed by blogs and forums before and after COVID-19 period, with some differences between diseases: blogs appeared as more relevant for heart attack compared to other diseases (Fig. 4A/B). Facebook is the main channel for sharing information (63%) (Fig. 4C).

### Analysis of reporter identity

We were able to identify the characteristics of the discussants regarding 758 conversations, as shown in Table 2. The identification of sex was possible only in a minor part of the study sample (Table 2). Across CVDs (Heart Attack, Heart Failure, Stroke) patients and caregivers were the most represented discussants. HCPs and patient associations increased their role during the COVID-19 period, especially answering to patient requests. Regarding heart attack conversations, the most represented discussants were mainly patients before the COVID-19 pandemic and again patients together with physicians during the COVID-19 period. Concerning heart failure conversations, the most represented discussants were mainly patients before the COVID-19 pandemic and patient associations and physicians during the COVID-19 period. More than half of the reporters in the pre-pandemic period who engaged in discussions about stroke were caregivers, whereas in the COVID-19 period they were both patients and caregivers. Health care professionals accounted for approximately a quarter (17.8–29.8%) of reporting for heart attacks and heart failure, but fewer health care professionals discussed stroke online (2.8% in the pre-pandemic period and 13.1% during COVID-19 pandemic). Overall, across the considered CVDs, health care professionals mostly contributed to the Web discussions mainly during the pandemic (Table 2).

**Table 2:** Characteristics of discussants according to cardiovascular disease type and timeframe

		**Pre-COVID-19**		**COVID-19**	
	**Characteristic**	**n**	%^*^	**n**	%^*^
Heart Attack	Male	8	*8.4*	13	*13.8*
	Female	8	*8.4*	3	*3.2*
	Unidentified	79	*83.2*	78	*83.0*
Heart Failure	Male	10	*6.8*	12	*6.8*
	Female	10	*6.8*	8	*4.5*
	Unidentified	126	*86.3*	157	*88.7*
Stroke	Male	39	*27.3*	38	*38.4*
	Female	44	*30.8*	45	45.5*
	Unidentified	60	*42.0*	16	16.2**
Heart Attack	Caregiver	10	*10.5*	14	*14.9*
	HCPs	23	*24.2*	28	*29.8*
	Patients	47	*49.5*	21	22.3**
	Groups/Organizations/Others	0	*0*	0	*0*
	Unidentified	15	*15.8*	31	33.0**
Heart Failure	Caregiver	21	*14.4*	21	*11.9*
	HCPs	26	*17.8*	51	28.8**
	Patients	35	*24.0*	30	*16.9*
	Groups/Organizations/Others	8	*5.5*	75	42.4**
	Unidentified	56	*38.4*	17	9.6**
Stroke	Caregiver	79	*55.2*	9	9.1**
	HCPs	4	*2.8*	13	13.1**
	Patients	47	*32.9*	60	60.6**
	Groups/Organizations/Others	0	*0*	0	*0*
	Unidentified	13	*9.1*	17	*17.2*

*P<0.05; **P<0.01 (chi-square test; pre COVID-19 vs. COVID-19)

### Analysis of cardiovascular topics of conversations

**Fig. 5:**
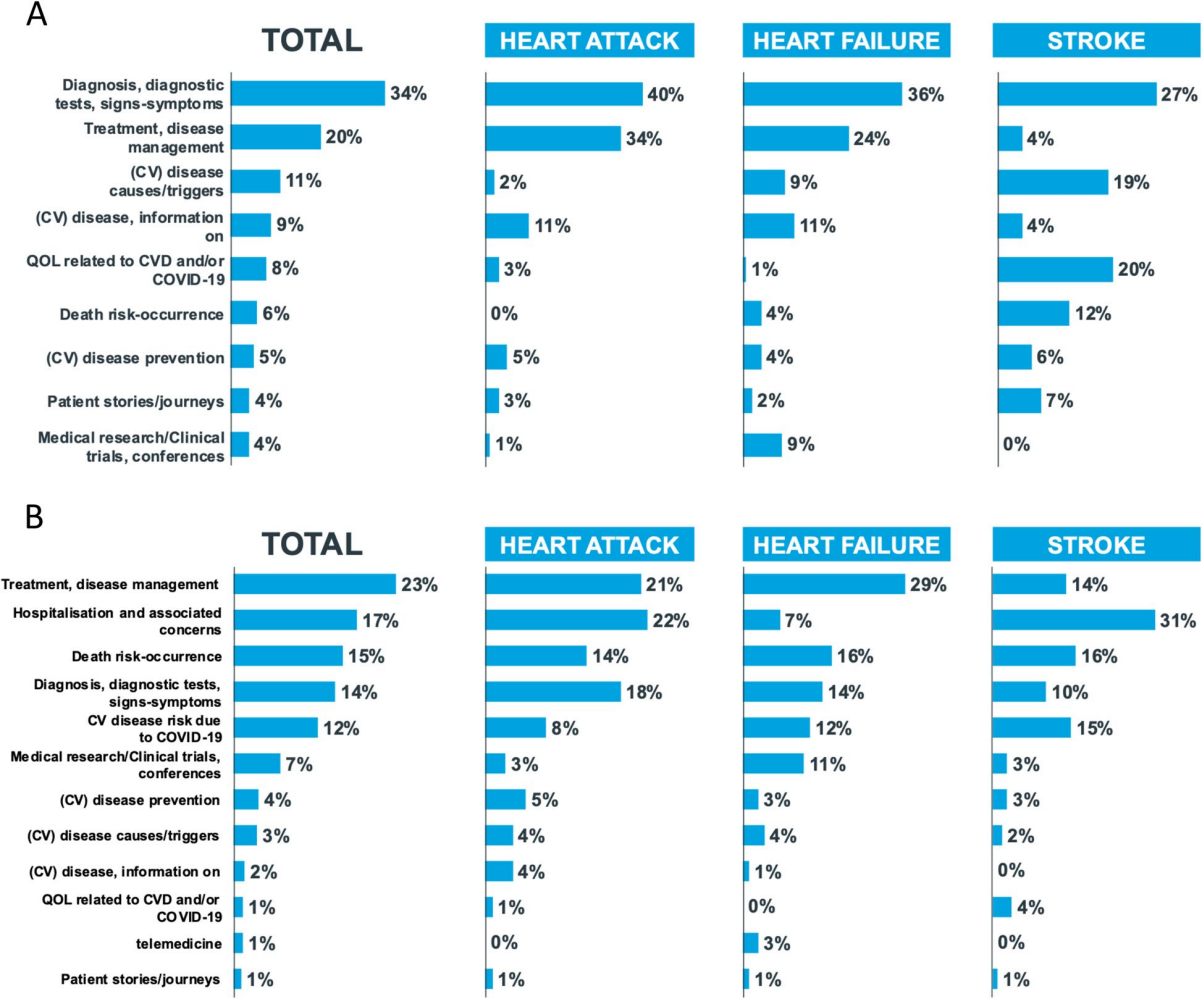
**A** Topics discussed during the pre-COVID-19 period: themes of discussions and their frequency during the pre-COVID 19 period across cardiovascular diseases. **B** Topics discussed during the COVID -19 period: themes of discussions during the COVID 19 period across cardiovascular diseases

The qualitative analysis of the conversations during the pre-COVID-19 period (Fig. 5A) shows that the main topics discussed about CVDs concerned symptoms and diagnosis (34%: respiratory problems and coughing as possible symptoms of heart failure, and chest pain and dizziness as symptoms of heart attack; dysphagia and tremor; electrocardiogram (ECG) for the diagnosis of heart attack and heart failure), treatments (20%: including discussions on stents and heart failure medications and on different types of drug therapy), causes and triggers of disease (11%: hypertension as trigger factor among patients with heart failure, heart attack and stroke; smoking as one of the main triggers for heart attacks and strokes; work stress as an important cause of arrhythmia, stroke and heart attack), disease information (9%) and quality of life (8%). Conversations on CVD prevention and lifestyle change (*i.e.*, correct nutrition and regular exercise programs) were marginal, scoring only 5% of the conversations. Conversation analysis shows significant differences across the different CVDs: discussions on quality of life, causes/triggers of disease, patient stories and death risk are more related to stroke; diagnosis and treatment more related to heart attack and heart failure, while clinical trials are more related to heart failure (Fig. 5A). Some practical examples of these conversations are collected in Table 1S.

During the COVID-19 pandemic, the topics of conversations included new themes, such as hospitalization and associated concerns (17%), death risk and occurrence (15%), CVD risk due to COVID-19 (12%), medical research and clinical trials (7%), and, to some extent, telemedicine (1%, rising to 3% for heart failure) (Fig. 5B). Conversely, a lower rate of discussions was relative to diagnosis and symptoms, disease causes and triggers, information on disease. Prevention of CVDs remained a marginal topic of discussion (4%). Discussions on fear about death risk occurrence were consistent across CVDs, hospitalization concerns were more associated to heart attack (22%) and stroke (31%), discussions on treatments and medical trials were more linked to heart failure (11%) (Fig. 5B). Some practical examples of these conversations are collected in Table 2S.

## Discussion

In most countries, there is a relevant cultural gap among citizens between optimal and current awareness about CVD prevention [3, 4, 6–9, 26]. As highlighted above, CVDs still represent the first cause of morbidity, mortality and disability [1]. The present study aimed to explore the awareness regarding appropriate CVD prevention and its implementation in one’s daily life within the Italian population. To achieve this goal, we selected the Web as the optimal source of information, since it represents an ecological space where people freely discuss, ask for information and get insights on health issues, as it has been clearly established. The qualitative and quantitative methods used in this study allowed us to understand health needs and concerns of patients, with the advantage of the absence of any bias due to external/observer interference [24–27].

This longitudinal study spanned across over more than 2 years, covering the 2 years before the COVID-19 pandemic and the first phase of the pandemic itself, including the lockdown phase, as a paradigmatic challenging phase, in Italy. This strategy allowed us to compare the health topics linked to CVD discussions during the 2 timeframes and to explore the impact of the early and most dramatic phase of the COVID-19 pandemic on the themes discussed. News channels and Twitter were found to be the most important platforms feeding information about CVDs, followed by blogs and forums. Facebook was instead the most relevant channel of information sharing. Before the COVID-19 pandemic, CVD ranked 5th among the main health issues discussed on the Web, following vaccines, tumors, influenza and diabetes, despite being the first cause of death. The 2-year timeframe before COVID-19 may represent the benchmark of the present study. Within this timeframe, the most discussed themes in the CVD field were, in decreasing ranking of relevance, symptoms and diagnosis (34%), treatments (20%), causes and triggers of disease (11%), information on disease (9%) and quality of life issues (8%). Conversations on CVD prevention were marginal, scoring only 5% of all conversations.

The abrupt occurrence of the COVID-19 pandemic and the related events, such as the lockdown, strongly impacted on the topics discussed, leading to the emergence of novel themes linked to this event, including, in decreasing ranking of relevance, hospitalization and associated concerns (17%), death risk and occurrence (15%), and CVD risk due to COVID-19 (12%). Prevention of CVDs remained a marginal topic of discussion (4%). The COVID-19 pandemic increased also patient fears linked to increased risk of severe COVID-19 among CVD patients and worsened the quality of relationship and contact with physicians, also due to difficulties to hospital access during the pandemic period. Indeed, this was actually reflected by a significant reduction of hospital admissions for acute myocardial infarction during the pandemic across Italy, with a parallel increase in related complication rates and fatalities [28, 29]. Moreover, a substantial increase of HCPs conversations, mainly answering to lay public questions, was observed, suggesting that reduced physical interactions increased patients and caregivers needs of information during the pandemic. Conversations about telemedicine occurred as a strategy to maintain contact with the physicians during the pandemic.

This study has some limitations, since the analysis of Web conversations, although conducted using rigorous and validated methodologies, can capture only discussions by the citizens who use the Web for their conversations, and not by the whole population, although most of the population itself may be even indirectly influenced by Web conversations. Moreover, the sex/gender of Web discussants could not be clearly identified, thus limiting this type of information, which actually is particularly relevant [30, 31]. Thus, since 57% of Italian population use the Internet to search information about health, the Web represents a substantial source of health information. A further level of complexity, that cannot be properly addressed in this type of studies, is the rapid change over time in the use of each social media, also according to sociodemographic features. Moreover, due to the intrinsic limitations associated with the analysis of Web conversations in Italy and in the Italian language, the findings of the present study cannot be extended to other Countries. In any case, similar approaches may be interestingly applied to other Countries, for future comparisons. This study highlights the value of Web listening through validated technologies as a reliable approach to assess the awareness of the general public in the health field and more specifically in the cardiovascular area. Furthermore, we had the opportunity to explore the quality and quantities of health-related Web discussions before and after a dramatic and unexpected event, the initial phase of the COVID-19 pandemic, which may be considered as a true divide for many human activities, including CVD prevention and management.

Our analysis showed that in Italy there is a limited overall attention to CVDs and, in particular, to their prevention. Additionally, patients and caregivers need more information and contact with their physicians: this gap dramatically emerged during the COVID 19 pandemic. What we registered through the conversations on the Web is once more not consistent with the important epidemiological relevance of such diseases in terms of health risks and mortality.

The results of the study highlight the urgency to promote novel prevention strategies and to engage people leveraging digital channels and social media. Interestingly, social networks have also been identified as potential tools for prevention and promotion of health, especially in younger subjects [25]. The occurrence of the COVID-19 pandemic increased patients fears about the impact of COVID-19 on CVD, the increasing risk of mortality and underline the problem of hospitalization, highlighting the difficulty to gain access to hospitals. It is then quite clear that policy makers and all relevant stakeholders in the health area should not just promote a better awareness about CVD among citizens and patients, but also support actions leading to the implementation of citizen/patient empowerment, possibly through digital solutions [11], acting on the same platforms from which we collected our data. Interestingly, some evidence of the effectiveness of education via social networking already started to be observed in randomized trials in patients with CVD [32]. Such evidence further emphasizes the need to develop novel models of patient management—even remotely (digital tools, telemedicine)—to encourage citizen/patient involvement and empowerment and to reinforce discussions on lifestyles and prevention. A practical and useful action following studies like the present one is to implement specific activities, based on the collected data and identified needs, operating through the same social media and aiming to increase the awareness of people on CVD prevention. Although a gold standard is not yet present in this area, one may first identify selected population targets according to age or other sociodemographic features and then define the best social media for that target. Afterwards, specific actions, to be defined, may be implemented, and their impact might be then measured through the same methods applied in this study. Moreover, involving young people as coresearchers in the development of specific interventions might be useful for designing a specific social network for health promotion for their range of age.

In addition, future research will extend the observations of the present study to the subsequent phases of the COVID-19 pandemic, taking into account the novel players in this field, like vaccines, which acted as important modifiers of health discussions in the general public, as well as the long-term effects, like the long-COVID syndrome.

## Conclusions

The analysis of Web discussions by validated methods showed that the Italian population has a very poor awareness about CVD, which ranks only 5th among the main health topics despite being the first cause of mortality. Information on CVD is mainly obtained from News channels and Twitter, while Facebook is more relevant for information sharing. The COVID-19 pandemic lockdown strongly impacted on the type of health topics discussed on the Web, without increasing awareness on CVD prevention. The observed overall limited awareness about CVDs and their prevention indicate the need to promote novel prevention strategies and engaging people in a more effective use of digital channels and social media.

## Supplementary Information

Supplementary Material 1.

## Data Availability

The datasets used and/or analysed during the current study are available from the corresponding author on reasonable request. The datasets were derived from sources in the public domain (Web-based social media).

## Abbreviations

CVD: Cardiovascular disease
ECG: Electrocardiogram
GBD: Global Burden of Disease
HCP: Health care professionals
NLP: Natural Language Processing
NCD: Noncommunicable disease

## References

[1] Collaborators GMaCoD. Global, regional, and national life expectancy, all-cause mortality, and cause-specific mortality for 249 causes of death, 1980–2015: a systematic analysis for the Global Burden of Disease Study 2015. Lancet. 2016;388(10053):1459–544. 27733281 10.1016/S0140-6736(16)31012-1 PMC5388903

[2] Roth G, Mensah G, Johnson C, Addolorato G, Ammirati E, Baddour L, Barengo N, Beaton A, Benjamin E, Benziger C, et al. Global burden of cardiovascular diseases and risk factors, 1990–2019: update from the GBD 2019 study. J Am Coll Cardiol. 2020;76(25):2982–3021. 33309175 10.1016/j.jacc.2020.11.010 PMC7755038

[3] Pelullo CP, Rossiello R, Nappi R, Napolitano F, Di Giuseppe G. Diabetes prevention: knowledge and perception of risk among Italian population. Biomed Res Int. 2019;2019:2753131. 31781605 10.1155/2019/2753131 PMC6875189

[4] Omboni S, Carabelli G, Ghirardi E, Carugo S. Awareness, treatment, and control of major cardiovascular risk factors in a small-scale Italian community: results of a screening campaign. Vasc Health Risk Manag. 2013;9:177–85. 23662063 10.2147/VHRM.S40925 PMC3646473

[5] Han CH, Kim H, Lee S, Chung JH. Knowledge and poor understanding factors of stroke and heart attack symptoms. Int J Environ Res Public Health. 2019;16(19):3665. 31569534 10.3390/ijerph16193665 PMC6801587

[6] Krishnamurthi RV, Barker-Collo S, Barber PA, Tippett LJ, Dalrymple-Alford JC, Tunnage B, Mahon S, Parmar PG, Moylan M, Feigin VL. Community knowledge and awareness of stroke in New Zealand. J Stroke Cerebrovasc Dis. 2020;29(3):104589. 31879136 10.1016/j.jstrokecerebrovasdis.2019.104589

[7] Hickey A, O’Hanlon A, McGee H, Donnellan C, Shelley E, Horgan F, O’Neill D. Stroke awareness in the general population: knowledge of stroke risk factors and warning signs in older adults. BMC Geriatr. 2009;9:35. 19656359 10.1186/1471-2318-9-35 PMC2734750

[8] Aminde LN, Takah N, Ngwasiri C, Noubiap JJ, Tindong M, Dzudie A, Veerman JL. Population awareness of cardiovascular disease and its risk factors in Buea, Cameroon. BMC Public Health. 2017;17(1):545. 28583117 10.1186/s12889-017-4477-3 PMC5460458

[9] Wang W, Zhang H, Lopez V, Wu VX, Poo DC, Kowitlawakul Y. Improving awareness, knowledge and heart-related lifestyle of coronary heart disease among working population through a mHealth programme: study protocol. J Adv Nurs. 2015;71(9):2200–7. 25879395 10.1111/jan.12668

[10] Chow CK, Teo KK, Rangarajan S, Islam S, Gupta R, Avezum A, Bahonar A, Chifamba J, Dagenais G, Diaz R, et al. Prevalence, awareness, treatment, and control of hypertension in rural and urban communities in high-, middle-, and low-income countries. JAMA. 2013;310(9):959–68. 24002282 10.1001/jama.2013.184182

[11] Pedretti RFE, Hansen D, Ambrosetti M, Back M, Berger T, Ferreira MC, Cornelissen V, Davos CH, Doehner W, de Pablo Y Zarzosa C, et al. How to optimize the adherence to a guideline-directed medical therapy in the secondary prevention of cardiovascular diseases: a clinical consensus statement from the European Association of Preventive Cardiology. Eur J Prev Cardiol. 2023;30(2):149–66. 36098041 10.1093/eurjpc/zwac204

[12] Siregar KN, Kurniawan R, BaharuddinNur RJ, Nuridzin DZ, Handayani Y, Retnowati, Rohjayanti, Halim L. Potentials of community-based early detection of cardiovascular disease risk during the COVID-19 pandemic. BMC Public Health. 2021;21(1):1308. 34217235 10.1186/s12889-021-11384-6 PMC8254668

[13] Duffy E, Chilazi M, Cainzos-Achirica M, Michos ED. Cardiovascular disease prevention during the COVID-19 pandemic: lessons learned and future opportunities. Methodist Debakey Cardiovasc J. 2021;17(4):68–78. 34824683 10.14797/mdcvj.210 PMC8588760

[14] Sabetkish N, Rahmani A. The overall impact of COVID-19 on healthcare during the pandemic: a multidisciplinary point of view. Health Sci Rep. 2021;4(4):e386. 34622020 10.1002/hsr2.386 PMC8485600

[15] Kentikelenis A, Ghaffar A, McKee M, Dal Zennaro L, Stuckler D. Global financing for health policy and systems research: a review of funding opportunities. Health Policy Plan. 2023;38(3):409–16. 36546732 10.1093/heapol/czac109 PMC10019567

[16] Roadevin C, Hill H. How can we decide a fair allocation of healthcare resources during a pandemic? J Med Ethics. 2021;47:e84. 10.1136/medethics-2020-106815 33441303

[17] Luciani S, Caixeta R, Chavez C, Ondarsuhu D, Hennis A. What is the NCD service capacity and disruptions due to COVID-19? Results from the WHO non-communicable disease country capacity survey in the Americas region. BMJ Open. 2023;13(3):e070085. 36863746 10.1136/bmjopen-2022-070085 PMC9990165

[18] Virani A, Singh G, Bewick D, Chow CM, Clarke B, Cowan S, Fordyce CB, Fournier A, Gin K, Gupta A, et al. Guiding cardiac care during the COVID-19 pandemic: how ethics shapes our health system response. Can J Cardiol. 2020;36(8):1313–6. 32505633 10.1016/j.cjca.2020.06.002 PMC7270812

[19] Sastry S, Cuomo F, Muthusamy J. COVID-19 and thrombosis: the role of hemodynamics. Thromb Res. 2022;212:51–7. 35219932 10.1016/j.thromres.2022.02.016 PMC8864963

[20] Xu S, Ilyas I, Little PJ, Li H, Kamato D, Zheng X, Luo S, Li Z, Liu P, Han J, et al. Endothelial dysfunction in atherosclerotic cardiovascular diseases and beyond: from mechanism to pharmacotherapies. Pharmacol Rev. 2021;73(3):924–67. 34088867 10.1124/pharmrev.120.000096

[21] Musa S, Dergaa I, Bachiller V, Saad HB. Global implications of COVID-19 pandemic on adults’ lifestyle behavior: the invisible pandemic of noncommunicable disease. Int J Prev Med. 2023;14:15. 37033280 10.4103/ijpvm.ijpvm_157_21 PMC10080576

[22] Rubio-Tomás T, Skouroliakou M, Ntountaniotis D. Lockdown due to COVID-19 and its consequences on diet, physical activity, lifestyle, and other aspects of daily life worldwide: a narrative review. Int J Environ Res Public Health. 2022;19(11):6832. 35682411 10.3390/ijerph19116832 PMC9180681

[23] Ye D, Pennisi S. Analysing interactions in online discussions through social network analysis. J Comput Assist Learn. 2022;38:13.

[24] Zhou L, Zhang D, Yang C, Wang Y. Harnessing social media for health information management. Electron Commer Res Appl. 2018;27:139–51. 30147636 10.1016/j.elerap.2017.12.003 PMC6105292

[25] García Del Castillo JA, García Del Castillo-López Á, Dias PC, García-Castillo F. Social networks as tools for the prevention and promotion of health among youth. Psicol Reflex Crit. 2020;33(1):13. 32671490 10.1186/s41155-020-00150-z PMC7363753

[26] Pengpid S, Vonglokham M, Kounnavong S, Sychareun V, Peltzer K. The prevalence, awareness, treatment, and control of hypertension among adults: the first cross-sectional national population-based survey in Laos. Vasc Health Risk Manag. 2019;15:27–33. 30881005 10.2147/VHRM.S199178 PMC6398413

[27] Reid E, Duffy K. A netnographic sensibility: developing the netnographic/social listening boundaries. J Mark Manag. 2018;34:23.

[28] De Rosa S, Spaccarotella C, Basso C, Calabrò MP, Curcio A, Filardi PP, Mancone M, Mercuro G, Muscoli S, Nodari S, et al. Reduction of hospitalizations for myocardial infarction in Italy in the COVID-19 era. Eur Heart J. 2020;41(22):2083–8. 32412631 10.1093/eurheartj/ehaa409 PMC7239145

[29] Cammalleri V, Muscoli S, Benedetto D, Stifano G, Macrini M, Di Landro A, Di Luozzo M, Marchei M, Mariano EG, Cota L, et al. Who has seen patients with ST-segment-elevation myocardial infarction? First results from Italian real-world coronavirus disease 2019. J Am Heart Assoc. 2020;9(19):e017126. 32901560 10.1161/JAHA.120.017126 PMC7792389

[30] Magni P. The sex-associated burden of atherosclerotic cardiovascular diseases: an update on prevention strategies. Mech Ageing Dev. 2023;212:111805. 37001567 10.1016/j.mad.2023.111805

[31] Mombelli G, Bosisio R, Calabresi L, Magni P, Pavanello C, Pazzucconi F, Sirtori CR. Gender-related lipid and/or lipoprotein responses to statins in subjects in primary and secondary prevention. J Clin Lipidol. 2015;9(2):226–33. 25911079 10.1016/j.jacl.2014.12.003

[32] Nouhi E, Dahesh T, Shojaefar F. Effect of media messages on health-promoting lifestyle of acute coronary syndrome patients: a randomized clinical trial. J Educ Health Promot. 2021;10:448. 35233395 10.4103/jehp.jehp_1457_20 PMC8827001

