# Supplementary material for: Web discussions on cardiovascular diseases: pre-COVID-19 evaluation and impact of the COVID-19 pandemic – Web listening analysis in the Italian population
Source: BMC Public Health. 2024 Nov 28;24:3316. doi: 10.1186/s12889-024-20615-5 (PMC11603844; doi:10.1186/s12889-024-20615-5)
Supplement: Supplementary file 1 — Supplementary Material 1. [file 12889_2024_20615_MOESM1_ESM.docx]

SUPPLEMENTARY MATERIALS

Table 1S – Examples of discussions (Pre-COVID-19 period)

| STROKE | Hi,my husband has had a stroke and ischemia, has inoperable carotids, occluded femoral etc. He is only 52 years old, we don't know what to do.  I am writing to you for comfort, but also advice, knowing that you are in the right place for having already visited this forum several times. My mom, 72, had an ischemic stroke following a coma caused by pneumonia. We were told that she would not have made it, and instead, despite having been very difficult, she managed to overcome the worst. For a couple of weeks, however, I have seen her very regressed, she no longer gives a speech and refuses even the slightest movements she previously made with enthusiasm. What can all this mean? Do you have experiences to tell me? Please help me. |
| --- | --- |
|  |  |
| HEART FAILURE | My 76-year-old mother, with mitral, tricuspidal and moderate aortic insufficiency, paps 50 mmHg, and FA, Parkinson's disease. For about 15 days she has had an annoying, mainly nocturnal cough, which greatly disturbs sleep. From June she takes ramipril, which she has stopped for about 6/7 days, but the cough persists. She also often experiences heartburn. She has no swelling in her feet. What causes coughing from cooking or gastric reflux? thank you. At the last visit, the cardiologist told me there is something but go quiet, everything is ok now, however, it is a week that I always tremble my hand and as soon as I make a minimum effort or a minimum effort, I tremble even more strong. And if I try too hard, my leg will tremble too (right side both hand and leg). I don't know what to do. Hi everyone.  Hi there. A respiratory crisis happened, months ago, to my mother, that after 5 hours led her to death. She had been in a small clinic for a few days. It begins in the evening, the beat at 100 with saturation at 80% and a lot of gurgling mucus, the nurse puts oxygen at 4 l / m. but nonetheless oxygenation does not rise. The doctor on call is also warned but the doctor himself is not alarmed. after 3 and a half hours the hot sweating also begins with breathlessness but with the usual sat 80% and beat at 100. The ambulance for the hospital was called after 3 hours and 45 minutes and will arrive after 35 minutes. At First Aid I was told she came with both 70% sat and cardiac arrest. (isn't it a contradiction !?). My mother was over 100 years old, had no health problems and did not take medicine. Entry for a few days. In hospital for dehydration she went out with head trauma and then sent to a small clinic. I would like to know what could have been the cause of death and if you see culpable behavior. Thank you.  Good morning, I find myself writing here for a problem that is affecting me. It's from Sunday that I feel like I'm missing my breath, it's not a real breath (like when running) but a continuous and urgent need to breath deeply and when it's not enough I resort to yawns which often I cannot even make them complete. I feel a sense of tightness in my chest and palate, I am all rigid and contracted. Let me start by saying that I have been suffering from this disorder since I was 9 years old, and it comes back to me every now and then, you just need to notice that I fall into this vicious circle. I performed 3 ECG in my life (as a newborn, at 12 years and 5 years ago, the last note made for this reason ... I thought you had a heart attack in progress). The problem is, I'm afraid of having heart disease or having lung problems. I am sporty, I never lack energy, I practice intense hiking in the mountains I am only heavily hypochondriac, in the past year I have gone (you can see my consultations) from ALS, MS, colon cancer, ovaries, brain ... now I'm obsessed with heart and lungs. What should I do? I asked the question to pneumologists but it also applies to cardiologists. I am very worried, I am afraid of having heart disease or having lung problems since birth. . . . I hope you can answer me, because I no longer remember how you breathe, I only have relief in my sleep. I was on a roller coaster on Saturday, could this have awakened a possible heart problem? |
| HEART ATTACK | Stopping smoking within one year of a heart attack is associated with a dramatic decrease in mortality in young adults. To say it is a study published on Jama Network Open, which showed how in a cohort of individuals (about half smokers) who had suffered a heart attack at a young age, those who quit smoking in the following year had rates of death from all causes and cardiovascular more than 70% lower.  Dear doctors, I am a 64 year old lady, after menopause I am on therapy with sartan Telmisartan 40 mg and beta blocker Nebivolol for hypertension and Omega 3 fatty acids (2 mg per day). I have grade I obesity, metabolic syndrome and hypertriglyceridemia. The cardiologist thought it appropriate to prescribe 100 mg of acetylsalicylic acid after lunch in primary prevention of stroke. I have no atherosclerosis, there have been no cases of myocardial infarction in the family and the cholesterol values ​​are as follows: total cholesterol 203 - HDL 31.6 - LDL 60. HDL cholesterol has lowered compared to a year ago. I would like to know politely if I need an antiplatelet as a prevention, or if losing about 10 kg and going for a walk I can avoid taking the drug and low HDL is associated with a risk of cerebral hemorrhage. Is this true and should I take fibrate as indicated to me? Thank you doctor.  Palermo is among the top ten cities in Italy with the highest mortality rate from acute myocardial infarction.  Good morning, I have been suffering from anxiety for years, since I had a sudden tachycardia with a sense of fainting, a feeling of not breathing and very fast beats for two hours. I take half a cup of paroxetine, every now and then I feel faint, chest pain, feeling of missing. Chest pain lasts for hours and I think I have reflux too. I'm worried about the sense of fainting. How to distinguish it from a heart attack or arrhythmia? When I go out of the pool after swimming I often turn my head a little bit, do I have to worry? Other times it happens to me at meals, especially towards the evening. Thank you doctor.  Every evening I have pain in the center of my chest and it lasts until I sleep .... anxiety, reflux or heart? Even today while swimming I had pain in the center of the chest, but no other symptoms. Thanks doctor. |

Table 2S – Examples of discussions (COVID-19 period)

| HEART ATTACK | COVID-19, risk of acute cardiac damage in children. Here's how statins are associated with a reduced risk of death among the elderly. Anticoagulants could improve the chances of survival for patients admitted to hospital with COVID-19. This is what emerges from a study published in the Journal of the American College of Cardiology, which according to the authors outlines an important therapeutic pathway for these patients.  Heart attack at 40. Hello Professor, in September 2019 after an hour of a cycling race I accused a strong pain of oppression in the chest and left arm. I went to the emergency room and I was found to have an acute myocardial infarction: angioplasty performed with stent insertion to the anterior interventricular branch, all for the best thank you all !!!  Since that day no one has been able to give me an answer on why those plaques obstructed the coronary artery even though I did not have a high cholesterol value, I have no family with heart disease, I have always led a sports life, and my age ... I am 42 years old !  I have heard conflicting opinions from various doctors (sports and non-sports) on returning to the sporting life of before and regaining eligibility for amateur competitions.  Am I a person at risk of relapse for this disease or can I consider it an unfortunate episode?  I would like, if possible, to have your opinion on this.  Thanks so much |
| --- | --- |
| Heart FAILURE | Good evening, 73 years old, for about 7 I have had three medicated stents - one of which is long - following therapy with Cardioaspirin 100, Diltiazem 60, Torvast 40. I suffer from severe bilateral gonarthrosis for which they are suggesting surgery, next summer. Total three-compartment prostheses.  Dear Mario,  A new publication that fills me with pride: "Pharmacological inhibition of GRK2 improves cardiac metabolism and function in experimental heart failure”.  A new study from the Icahn School of Medicine in New York has just revealed that people who eat a diet high in fruit and vegetables are 42% less likely to develop heart failure than those who consumed fewer plant-based foods.  French hospital halts trials of Trump-promoted COVID-19 drug due to worries about heart failure.  Top stories Yale patient registry provides a connection between heart failure and COVID-19  I had a wake-up call last night. My body went into convulsions I couldn't talk or walk. because I am in stage 4 heart failure the ambulance people could not take me to hospital. they did a video link with doctors. thanks to the wonderful ambulance and docs I'm ok.  Estimates Feasible for Heart Failure Telemedicine  My mother-in-law went into hospital end Feb with kidney & heart failure. She passed away just under a week ago. Spoke to Registrars office this morning who tell us that COVID-19 is mentioned on death certificate.  My cousin died in Harare of heart failure after being on incorrect medication for a year. On his last day, Harare hospital was closed. The private clinic demanded USD500 & a COVID-19 certificate no more than 2 weeks old. Only hospitals are testing. He died on his way back home.  The AFMPS announced that the EMA advised against interruption in the use of medicinal products for treatment of hypertension, heart failure, or kidney disease despite reports that the use of these products may aggravate COVID-19 infection.  The use of beta-blockers to treat hypertension appears to increase the risk of heart failure among women with acute coronary syndrome (ACS), according to a new analysis published in 'Hypertension'.  16.9% of people that died due to COVID-19 infection had an history of heart failure before being infected". Not all heart failures are lethal. |
| STROKE | Hi,my husband has had a stroke and ischemia, has inoperable carotids, occluded femoral etc. He is only 52 years old, we don't know what to do.  I everyone, I'm 29 and have been smoking 2-3 cigarettes a day (during the week) for almost a year and nothing on the weekend. I often have pains on the left (above and below the breast, in the arm and shoulder blade) and in the center of the chest. Sometimes with paresthesias. I am an anxious person (I suffer from panic attacks from time to time and I take Xanax as needed) and when I get these pains I panic and think I have a heart attack. I have anyway after not a little effort (I stopped and started a few times) to stop definitively from today. I smoked my last cigarette today and threw the pack away. I wanted to know what you think of the symptoms reported and especially how much I am at risk of heart attack considering that I have definitively stopped since today. Yesterday I had a headache all day. I thought it was a stroke, but luckily I'm still here. Looking forward to your kind replies.  Doctor I wanted to ask you if this ischemia I have had is a transient ischemic attack or not ??! What should I expect? I read that it is a year that I should have a stroke. But I take cardioaspirin, could I still have it? Then another thing. The fact that I always have a headache especially the right part I discovered that I have ischemia is it due to ischemia? Thanks a lot for the answer.  Bad news for deniers. Coronavirus, goodbye to Canadian actor Nick Cordero. He had been hospitalized at Cedars-Sinai Hospital in Los Angeles, California for over 90 days, and doctors had given him mechanical ventilation, after a mini-stroke, a series of blood clots and infections. He had been implanted with a temporary pacemaker, had his right leg amputated, had a tracheotomy and the doctors were also considering the possibility of a double lung transplant. but it's okay, the #virus that disappeared #no one gets sick anymore. COVID-19 disease leaves effects of varying severity. It does not heal at 100%, during the acute phase it causes a lot of systemic damage in particular to the cardio-respiratory system.  Strokes, heart attacks, respiratory failure, kidney failure are the damage we have to deal with once the virus passes. |
